# Microbial population dynamics during traditional production of Mabisi, a spontaneous fermented milk product from Zambia: a field trial

**DOI:** 10.1007/s11274-020-02957-5

**Published:** 2020-11-16

**Authors:** Anneloes E. Groenenboom, John Shindano, Nachimuka Cheepa, Eddy J. Smid, Sijmen E. Schoustra

**Affiliations:** 1 Laboratory of Genetics, Wageningen University and Research, Wageningen, The Netherlands; 2 Food Microbiology, Wageningen University and Research, Wageningen, The Netherlands; 3 Department of Food Science and Nutrition, University of Zambia, Lusaka, Zambia; 4 Heifer International Zambia, Lusaka, Zambia

**Keywords:** Bacterial community, Dairy, Fermentation vessel, Field study, Traditional fermentation

## Abstract

Mabisi is a fermented milk product, traditionally produced in a calabash by uncontrolled fermentation. Due to high costs and a reduced availability of calabashes, nowadays plastic containers are also used for Mabisi production. However, the effect of this change in production practice on the properties of the product has not been documented. Therefore, we aimed at determining the effect of fermentation vessels and types of back-slopping on acidification and microbial communities during fermentation. A series of fifteen experiments using two types of fermentation vessels (plastic buckets and calabashes) in combination with different types of back-slopping (no back-slopping, passive back-slopping, and active back-slopping) were set up at a field site in rural Zambia. In each of the fifteen fermentations we analysed acidification rate of traditional Mabisi fermentation and bacterial diversity over time. No significant difference was found in terms of microbial diversity during and at the end of fermentation between fermentations performed in buckets or previously used calabashes. Bacterial communities in general decreased in diversity over time, where the drop in pH correlated with a decrease in Shannon Index. In case of active back-slopping, the pH drop started right after inoculation while in the no back-slopping and passive back-slopping fermentations, there was a clear lag phase before acidification started. All experimental series resulted in a microbial community dominated by *Lactococcus lactis* and a Shannon Index, as a measure for diversity, between 0.6 and 2.0. The use of plastic buckets for Mabisi fermentation can be a valuable alternative to the use of calabashes as this study showed no biological and physico-chemical differences between Mabisi resulting from both fermentation vessels, although the reason for perceived differences should be further investigated.

**Electronic supplementary material:**

The online version of this article (10.1007/s11274-020-02957-5) contains supplementary material, which is available to authorized users.

## Introduction

Traditional products made by fermentation are an important aspect of culture, nutrition and livelihoods in many countries. Mabisi is such a product found in many rural areas in Zambia (Chileshe et al. 2020a; Moonga et al. 2019). Mabisi is fermented cow milk traditionally produced in a calabash by uncontrolled fermentation that is dominated by six to ten different species of lactic acid bacteria (Schoustra et al. 2013). Households have their own calabashes that they use for consecutive rounds of Mabisi production. The methods of producing Mabisi vary depending on location and can include back-slopping, churning, and other production methods (Moonga et al. 2019). The ‘Tonga-type’ is the most simple and commonly practiced way of producing Mabisi, in which milk is sieved before allowing it to incubate statically at ambient temperature for 24 to 48 h in a calabash and is stirred right before consumption. The moment of consumption is determined by organoleptic observations: when the Mabisi is thick and sour enough it is ready for consumption (Moonga et al. 2019).

Due to high costs and a reduced availability of calabashes, nowadays more and more plastic and metal containers are used for Mabisi production. The main process remains unchanged: fresh raw milk is collected in the container and left to ferment for 24 to 48 h at ambient temperatures. Although this method is accepted nationwide, there is the general belief that milk fermentation in calabashes results in a Mabisi with better organoleptic characteristics than Mabisi produced in plastic containers (Moonga et al. 2019). This perceived difference might be caused by differences in the bacterial communities present in the fermenting milk (Schoustra et al. 2013). These fermenting bacteria will form most of the volatile aroma compounds and are responsible for other specific product attributes such as viscosity, which together determine the flavour and overall organoleptic properties of the Mabisi. A more diverse microbial community might result in a product with a richer flavour palate as it is likely to produce a higher variety of aromas (Dertli and Çon 2017; Randazzo et al. 2006; van Rijswijck et al. 2017) and it increases the chance of high flavour forming strains being present (Fernández de Palencia et al. 2006).

In spontaneous fermentation such as in the production of Mabisi, bacteria can enter the raw milk and start the fermentation from various sources. As the milk is not pasteurised, all bacteria that were already present or entered the milk via the air or the milking equipment used, will still be present at the onset of fermentation. This can be the case in both types of fermentation vessels, calabashes and buckets. A key difference, however, is the inner surface of both vessel types. Where buckets have a smoother surface, which can be washed and dried more easily between fermentation cycles, the calabashes are made of natural material and have a rough inner surface. The calabash surface provides a great opportunity for bacteria to form biofilms and hide in small cracks without being removed during washing and drying. Most likely, the bacteria that remain in the calabashes after a previous fermentation cycle are essential for starting a new fermentation cycle (Groenenboom et al. 2019a). Without this rough surface, fermentation using buckets might result in a less diverse microbial community and therefore in a less rich product. Back-slopping might increase the diversity in the buckets to a level comparable to calabashes. Here, we define two types of back-slopping: (1) active back-slopping, where finished product is added to the raw milk to start the fermentation, and, (2) passive back-slopping, meaning the un-intentional transfer of bacteria between two rounds of fermentation by using the same fermentation vessel.

Knowledge is lacking on the influence of fermentation vessels and types of back-slopping on the spontaneous fermentation of Mabisi in Zambia. Therefore, a field study was conducted in rural Zambia to determine the influence of the two specific variables on the rate of acidification and on the microbial community present in the final product of Mabisi: (1) type of fermentation container used, a plastic bucket or a calabash, and, (2) type of back-slopping between different rounds of fermentations, no back-slopping, active or passive back-slopping. Since we used traditional methods of spontaneous fermentation, several variables were uncontrolled, such as temperature and batch volumes. We nevertheless monitored these uncontrolled variables, to gain insight in the potential effects of these variables on the overall fermentation trajectory and specific characteristics of the fermentation process.

## Materials and methods

### Field site

Experiments were performed at the Mondake Dairy Association based in the rural part of Kabwe town in Zambia (14°19′09.1″S 28°39′59.1″E). Local famers in this region are supported by Heifer International (Zambia), an NGO that provides dairy cows and training in animal husbandry as one of the means to alleviate hunger in rural areas. More information about the local community can be found in “Appendix B”.

### Experimental setup in the field

The experiments were conducted in July 2015 and more information about the field experiment can be found in “Appendix Fig. A”.

In total, we performed fifteen fermentation cycles, producing Mabisi from raw milk (Table 1). Three ways of back-slopping were applied (no back-slopping, passive, and active back-slopping) in two types of fermentation vessels (calabashes and plastic buckets). Passive back-slopping in buckets was considered to be not significantly contributing to transfer of bacteria between fermentation cycles, as the buckets were thoroughly cleaned with water and soap after every fermentation round. This resulted in five different treatments for fermentation (Table 2). The availability of the equipment (fermentation vessels and pH and temperature probes) and the amount of milk, caused the experiments to be conducted over the course of 14 days. Five fermentations could be monitored simultaneously in terms of temperature and pH.

**Table 1:** Details of all fifteen fermentations

	Date	Time	fermentation vessel	Back-slopping	Volume (L) total/in vessel		T and pH measured
1	16 July	13:40	Calabash	Passive	2.5	2.5	Yes
2	17 July	14:40	Bucket	No	6.5	3.25	
3			Bucket	Active		3.25	
4	18 July	15:00	Calabash	Passive	3.5	3.5	
5	19 July	16:15	Calabash	Active	9.5	9.5	
6	20 July	15:30	Calabash	No	11	11	
7	21 July	15:15	Calabash	Active	23	7.77	
8			Bucket	Active		7.67	
9			Bucket	No		7.66	
10	22 July	16:30	Calabash	Passive	8	8	
11	23 July	15:30	Bucket	No	7	7	
12	27 July	13:00	Calabash	Active	18.5	4.63	No
13			Bucket	Active		4.62	
14			Bucket	No		4.63	
15			Calabash	Passive		4.62	

Time indicates moment at which milk was poured into the fermentation vessels. This field experiment was performed in 2015

**Table 2:** The summary of conditions of all fifteen fermentations

	Not back-slopped	Passively back-slopped	Actively back-slopped
Calabash	1	4	3
Bucket	4	–	3

Fermentation are performed in calabashes or buckets as fermentation vessel and back-slopped or not. An unequal number of fermentations per treatment was caused by: (1) One new calabash was available to perform a not back-slopped fermentation. (2) Passive back-slopping is not considered possible in plastic buckets due to the smooths surface on the inside of the bucket. (3) Due to time constrains two fermentations with active back-slopping could not be performed

Three calabashes (about 20 L capacity) were obtained from local markets, two of the calabashes were provided to the Mabisi producers two months before the start of the study to be used for Mabisi production. One calabash had never been used for Mabisi production before the study, which made it possible to perform one non back-slopped fermentation in that vessel type. The calabashes used for all other fermentations had been used before and were therefore considered to be a source of inoculum for the next fermentation cycle via passive back-slopping. According to traditional practice, calabashes were all filled with water and a mix of grass and cane, and left for a day to prepare them to be used for Mabisi fermentations. Between fermentation cycles, the calabashes were washed with water. All water used was untreated water from a small stream, according to common practice and availability. Three white buckets with lids (20 L capacity) were bought from a nationwide grocery store and brought to the field site. Buckets were cleaned with water before and after fermentation rounds.

**Fig. 1:**
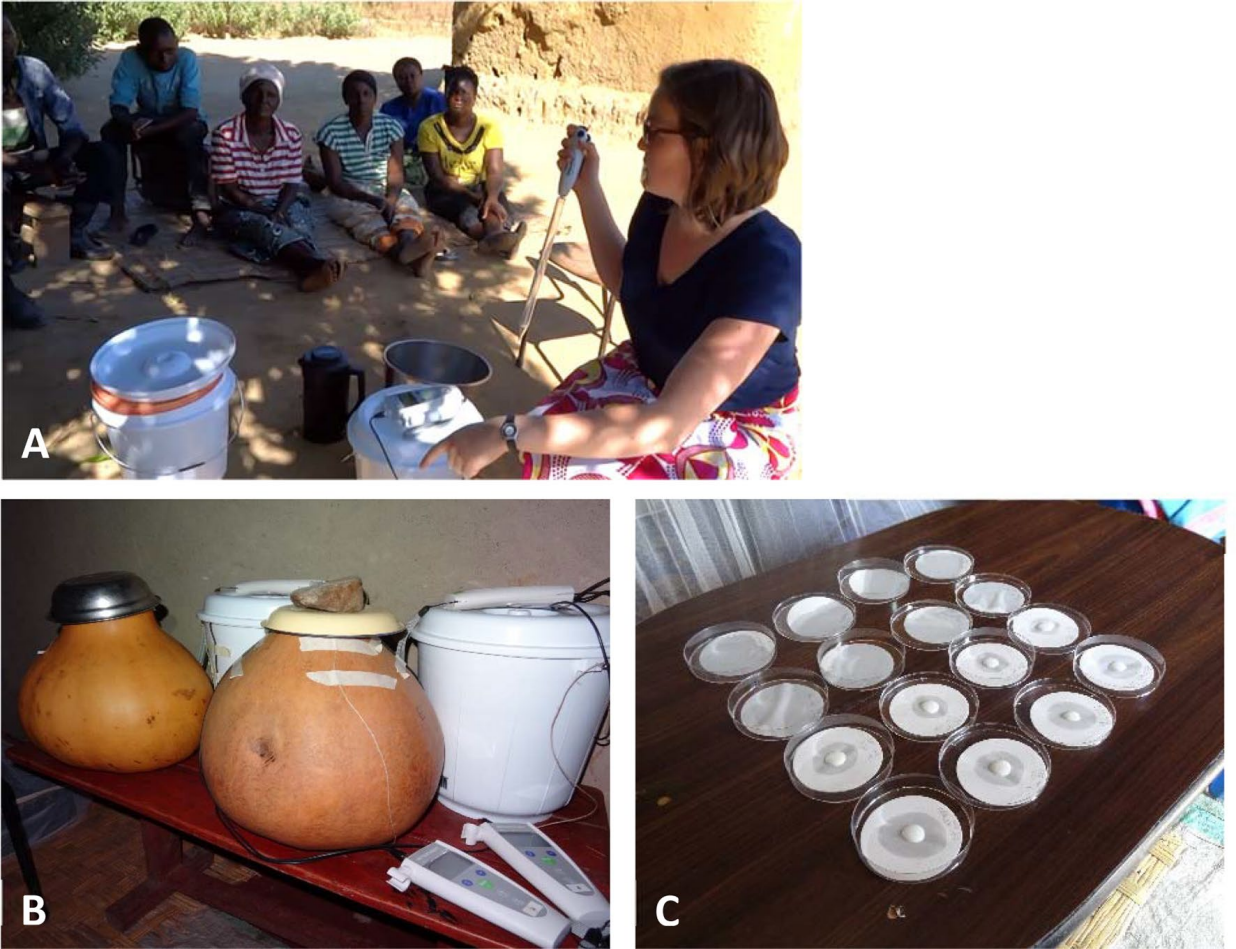
Set-up of the field-experiments. Panel **a** In the afternoon the fermentations were started. Active back-slopping was performed using a pipette. The local community showed great interest in the study. **b** Fermentation was performed in calabashes and plastic buckets, with pH meters and temperature probes attached. At night the containers were put inside the house to prevent temperature drops. **c** Samples of Mabisi at different stages of fermentation were stabilised on a filter paper

Fermentation vessels were equipped with temperature probes (Thermochron High resolution 15/46 °C, Sydney, Australia) in water tight capsules (Thermochron, Sydney, Australia) and pH-probes (FiveGo, Mettler Toledo, Schwerzenbach, Switzerland), continuously placed in the fermenting medium (Fig. 1b). The starting time of fermentation was dependent on the milking time of the farmers and was typically between 1 and 4PM. For active back-slopping, the absolute amount of finished Mabisi was dependent on the volume of the new fermentation; the fresh milk was inoculated with 1% (v/v) finished Mabisi at the start of a new fermentation cycle. For passive back-slopping and no back-slopping no further actions were taken. During the day the fermentation vessels were positioned outside in the sun or in the shade. During the night the containers were stored in a hut, which was heated by hot coals.

As expected of field experiments that involve local processors, ours was not without limitations and we took note of all the possible limitations as summarized in Table 3 which shows all (un)controlled and (un)measured variables. The milk production of the cows was variable, as well as the availability of the farmers that would milk the cows and deliver the milk to the field location. Milk was obtained from up to six farmers, but mostly three farmers. The milk was combined into one batch and sieved right before it was put in the fermentation vessels. As all the morning milk was collected to be sold to a nationwide operating dairy company, only the afternoon milk was used for this study. To prevent food loss, all afternoon milk that was made available was used for the fermentations, which resulted in variations in fermentation volumes (Table 1). As local customs and traditions were taken into account, and in keeping with how the fermentation is exactly conducted in the community, also the starting time of fermentation and the sampling regime varied per fermentation. Only one new calabash was available, and therefore only one no back-slopping experiment could be set up for this vessel type.

**Table 3:** Variables which were (un)measured and (un)controlled during the field fermentations

	Unmeasured variables	measured variables
Uncontrolled variables	Time between milking and ‘start’ of fermentation	Temperature during fermentation cycle
		Fermentation volume
	Cows from which the milk originated	Start time of fermentation
Controlled variables	–	Fermentation vessel type
		Back-slopping method
Output variables	–	pH Microbial community composition during fermentation

### Sampling of Mabisi for microbial analyses

For the first eleven fermentations (Table 1) samples of the fermentation liquid were taken during fermentation using sterile pipets (Greiner Bio-One, Frickenhausen, Germany) and a Smoothie pipet filler (VWR/Avantor, Radnor, USA). Sampling times were typically at 0 h, 0.5 h, 2 h, 6 h, 16 h, 20 h, 24 h, 40 h and 48 h after the start of fermentation, but varied per fermentation cycle due to circumstances of the field site (such as cultural obligations and night time). These samples were stabilised on filter papers as described in Groenenboom et al (2019a, b) (Groenenboom et al. 2019b) to allow analysis of microbial community composition (Fig. 1c). All samples were subsequently air shipped to the Laboratory of Genetics at Wageningen University, the Netherlands for DNA extraction (Ercolini et al. 2001; Groenenboom et al. 2019b). The extracted DNA is the starting material for the bacterial community analyses.

### Analyses of bacterial communities

The extracts containing DNA from all organisms in the microbial community were sent for bacterial 16S rRNA gene amplicon paired-end sequencing of the V4 hypervariable region (341F-785R) on the MiSeq Illumina platform by LGC genomics (Berlin, Germany).

For further data processing and statistics the QIIME pipeline (Caporaso et al. 2010), modified from Bik et al (2016) was used (Bik et al. 2016). Paired-end reads were joined using join_paired_ends.py (with minimum overlap 10 base pairs) after which sequences were trimmed and filtered using cut adapt (v1.11-q 20, -m 400) and using the known primer sequences CCTACGGGNGGCWGCAG and GACTACHVGGGTATCTAAKCC to trim both sides of the sequence. These trimmed sequences were then checked for chimera’s, using UCHIME (v4.2.20, gold database (Edgar et al. 2011); sequences with a chimera score lower than 0.28 were retained. Next, sequences were clustered into operational taxonomic units (OTUs) after quality check using pick_open_reference_otus.py (-s 0.1, -enable_rev_strand_match TRUE, -align_seqs_min_length 75, -pick_OTU_similatiry 0.95). Taxonomy of the resulting OTUs was assigned to representative sequences using the Green genes (v13.5) rRNA database. This algorithm gives a representative sequence for an OTU, which were used to perform a local BLAST using the gold database from uchime (Altschul et al. 1990). Shannon index (H) accounts for both number and evenness of OTUs present and is calculated using: $H =-{\sum }_{i=1}^{s}\left({p}_{i}\mathrm{ln}{p}_{i}\right)$ in which p_i_ is the proportion of reads belonging to category i, and s is the total number of categories which can be OTUs, species, or genera depending on the level of clustering of the reads. The Shannon index can lie between 0 and ln s (Hutcheson 1970), depending on the distribution of the categories (evenness). The number of identified OTUs (s) in Mabisi communities ranges from about 40 (typically at the beginning of fermentation) to 400 (typically at the end of fermentation) per sample, giving a maximal Shannon index between 3.7 and 6.0 (ln s). Pairwise Spearman’s correlation of Shannon index values and pH was estimated for samples obtained using filter paper method (Groenenboom et al. 2019b).

## Results

### Documentation of uncontrolled variables

**Fig. 2:**
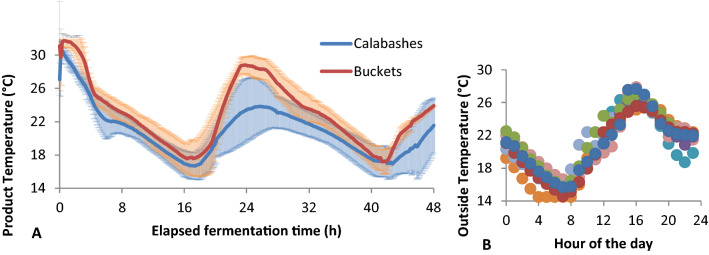
Temperature during fermentation **a** Temperatures measured using a temperature button in the milk during fermentation. Lines show averages of all fermentations using a calabash (blue line) and a bucket (red line). Error bars indicate standard error from the average calculated over 6 fermentations in calabashes and 5 fermentations in buckets. Start of fermentation (t = 0 h) happened at different time of the day for all fermentation but was typically between 1 and 4PM. **b** Daily air temperatures measured inside the hut where the fermentation vessels were stored at night for the period between 16 and 27th July 2015. Different colours indicate different days. The temperature buttons could not measure below 14 °C

**Fig. 3:**
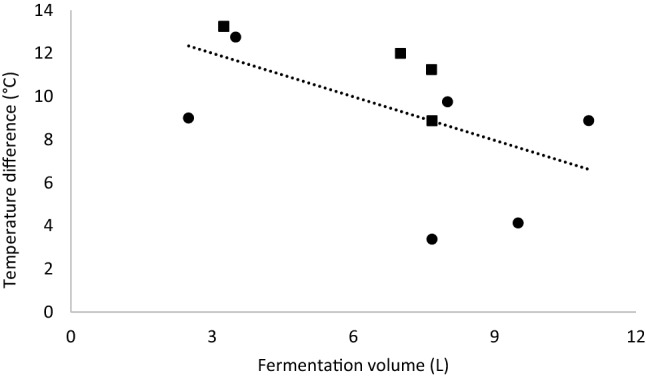
Temperature change as a function of volume of milk used for fermentation in calabashes (filled circle) and buckets (filled square). Temperature change is the difference in temperature between the minimal (around 16 h after the start of fermentation) and maximal (around 24 h after the start of fermentation) measurement during the fermentation cycle. Pearson correlation: r =  − 0.568, p = 0.068

Ambient temperatures in July in Zambia are quite predictable. Normal outside day temperatures are in the range of 22 to 25 °C (Fig. 2b). The fermentations were performed outside during the day and inside during the night, allowing temperatures to increase under the influence of sunlight during the day and tempering the cooling down during the night when outside temperatures drop to as low as 5 °C. The product temperatures in the fermentation vessels are not only influenced by the outside temperature but also by vessel type and volume of the fermented milk. The fermentation vessels used have a different shape and are made from different material, resulting in differences in isolation and the ability for evaporation. On average the temperature of the milk in buckets increased at a faster rate and reached a higher level than the milk in calabashes (Fig. 2). During the night the milk in both fermentation vessel types cooled down to a similar level. After around 24 h of fermentation the difference in milk temperature between the two types of fermentation vessels was the largest. Apart from the vessel type, the volume of the milk can affect the temperature profile. The average volume in calabashes was a bit higher than the average volume in the buckets: 7.0 ± 3.1 L and 5.8 ± 2.1L, respectively. When we plot the maximum temperature difference per fermentation as a function of volume, we see a non-significant negative correlation (Fig. 3).

### Acidification

**Fig. 4:**
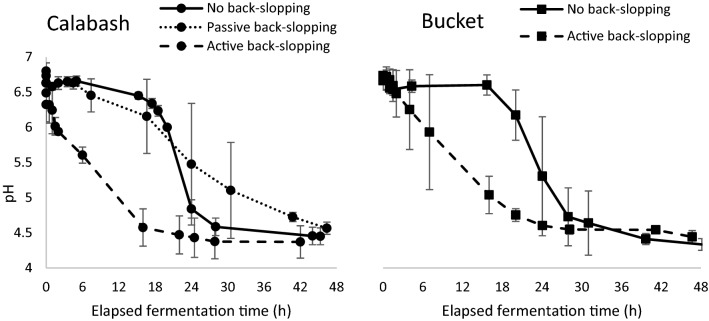
pH over time during fermentation with no back-slopping and passive and active back-slopping in calabashes and no back-slopping and active back-slopping in buckets

The pH was measured during all fermentations in the five different treatments (Table 1). Results are given in Fig. 4 and showed that in all cases the pH had dropped below pH 5 after 48 h. The time elapsed before the pH dropped below pH 5 varied. When a new calabash or a new or not back-slopped bucket was used, the drop in pH as a measure for fermentation activity had a lag time of about 18 h. This lag time was not present in the fermentations that were actively back-slopped; the pH decreased below 5 within 18 h. In case of passive back-slopping due to the re-use of a cleaned calabash the results were mixed; in one case the lag time was even longer than 18 h while in other fermentations the pH was below 5 within 24 h.

### Microbial community composition

**Fig. 5:**
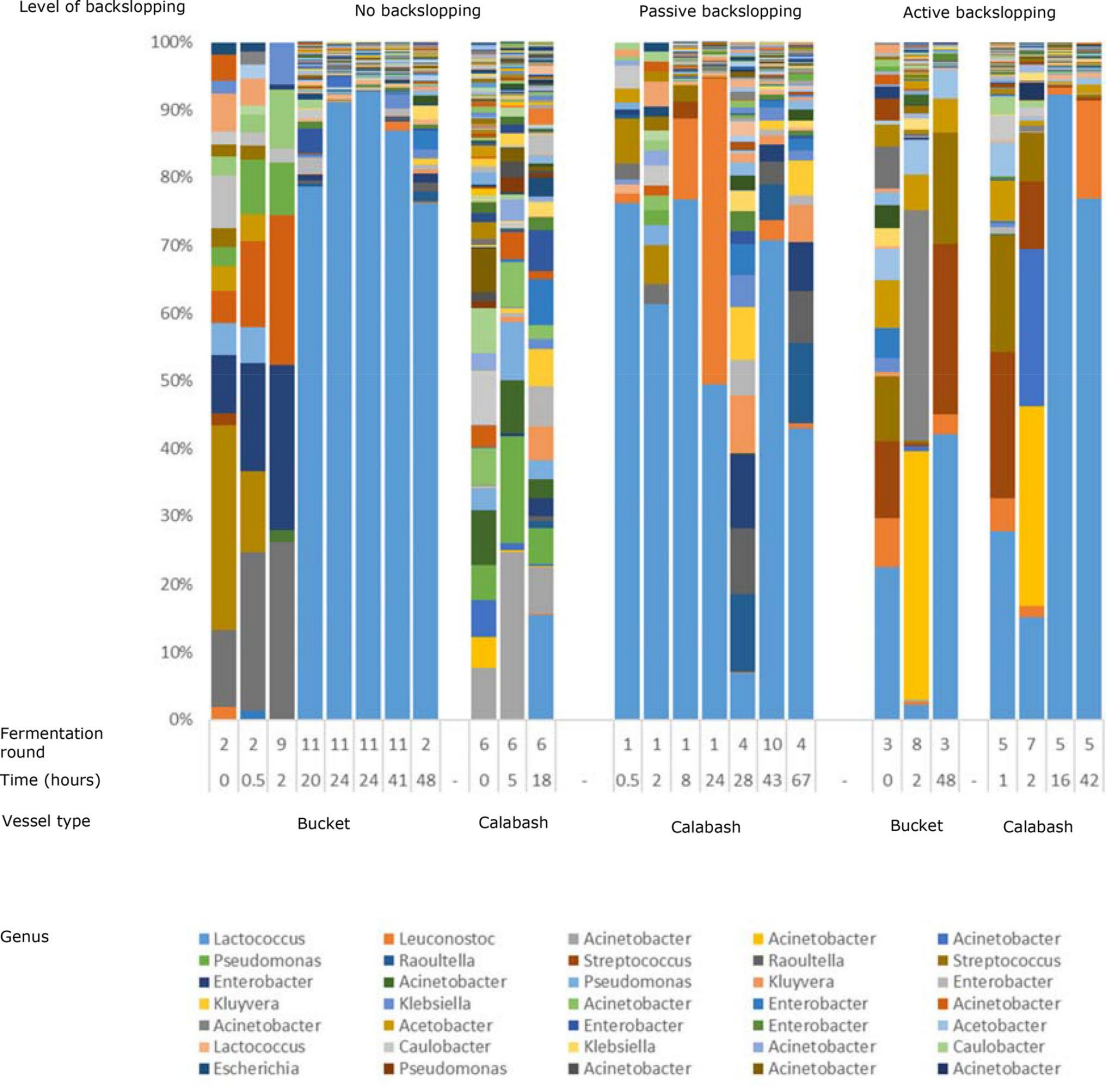
Bacterial community structure of representative Mabisi samples during the course of fermentation using different levels of backslopping. Different colours represent different operational taxonomic units (OTUs). Total number of OTUs per sample was taken as 100%. BLAST results to the genus level of the most abundant 21 OTUs are given. *Fermentation round, details in Table 1. **Time after start of fermentation in hours. Some samples could not be included because the field experiment did not allow regular sampling and some samples were lost due to contamination. This figure shows a representative subset of all samples that were analysed. The results of all samples are in Online supplementary material 1

Samples were shipped to the laboratory in the Netherlands where DNA was extracted. The extracts were sent for 16S amplicon sequencing and the resulting reads were analysed to construct bacterial community compositions. Figure 5 shows a representative subset of samples; all bacterial community profiles analysed are in Online Supplementary material 1. On average the most abundant species were found to be from the genera *Lactococcus, Leuconostoc, Streptococcus, Acinetobacter* and *Acetobacter*. The most abundant OTU (between 43 and 96% of all OTUs) returned as a *Lactococcus* strain from the BLAST analysis. This OTU was most abundant in all samples towards the end of fermentation (after 40 h or more) for both buckets and calabashes. In samples taken at the beginning of fermentation (within the first 5 h) more diverse bacterial communities are found containing various genera that were not found at later time points of fermentation, such as *Mycoplasma* and *Clostridium* species.

**Fig. 6:**
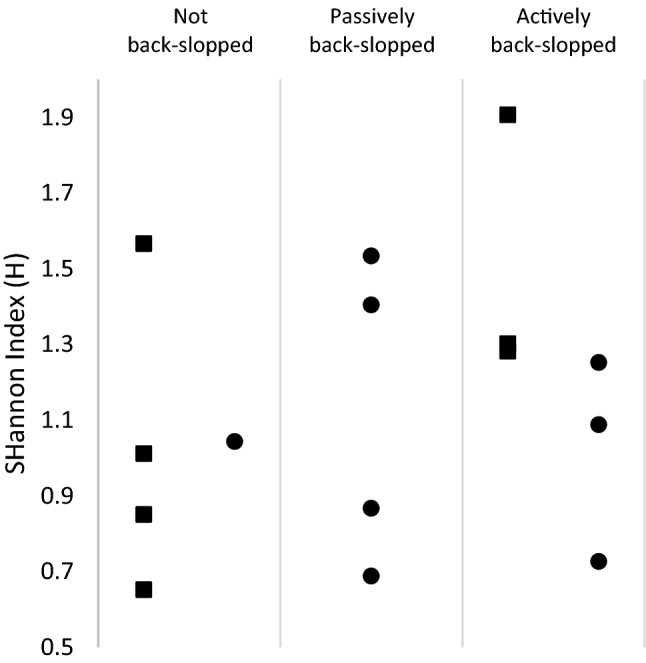
Shannon Index of microbial communities at the end of fermentation in calabashes (filled circle) and buckets (filled square). Only one fermentation was performed in a new calabash and due to the smooth inner surface, the possibility for passive back-slopping in buckets was neglected. No significant differences between fermentation could be found, possibly due to lack of statistical power

The Shannon index of the bacterial communities was calculated as a measure for community diversity (“Appendix Fig. C”). New calabashes stand out in that they harbour microbial communities of higher diversity than observed in the other treatments of used calabashes or buckets. Furthermore, there is no difference in diversity when different ways of back-slopping were used. The end-point samples obtained from all fifteen fermentations provide an overview of the results of all five methods (Fig. 6). No difference in final community structure was found between different ways of back-slopping or the use of fermentation vessel. Considering active back-slopping we observed a trend towards a higher microbial diversity in the buckets compared to the diversity found in calabashes. In buckets, active back-slopping appeared to increase the communities’ bacterial diversity compared to bacterial communities of fermentations without back-slopping.

**Fig. 7:**
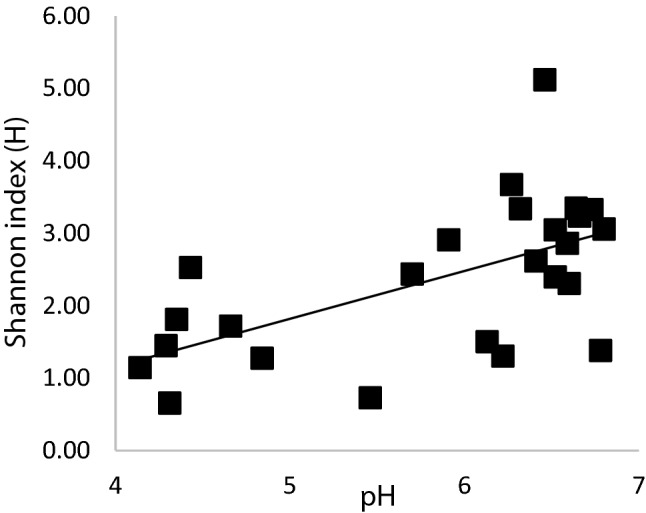
Shannon index (based on OTU) plotted as a function of pH indicated a significant positive correlation between the two. The pH lowered as the fermentation progressed which led to a lower species diversity (Spearman’s correlation coefficient = 0.582, df = 23, p = 0.002)

Interestingly, at a lower pH the overall diversity of a community was lower (Fig. 7). This correlation was uncovered using all available samples obtained via the filter paper method during fermentation. Although the acidification rate in the different fermentation methods had different rates, this clearly correlates with the community diversity.

## Discussion

This study focussed on the spontaneous fermentation of Mabisi using traditional calabashes and plastic buckets. The overall objective of this study was to assess the effect of variations in type of fermentation vessel used and in the type of back-slopping on the pH drop and microbial community dynamics during fermentation cycles. In case of fermentations in calabashes the effect of passive back-slopping, where back-slopping occurs unintentionally through the transfer of bacteria via the inner surface of the vessel, was considered. Temperature, batch volume, and timing of the start of fermentation during the day were uncontrolled factors, which effects were investigated as well.

### The effects of uncontrolled factors

The uncontrolled factors are interrelated. The time of day at which the fermentation was started had an implication for the temperature of the milk during fermentation. Temperature during fermentation is mainly dependent on the outside temperature; during the night the temperature of the milk drops below 18 °C and during the day temperatures can rise to values above 30 °C. There are indications that fermentation vessel and the fermentation volume also had an influence on the temperature profile during fermentation. In calabashes we observed a lower maximum temperature compared to the maximum temperature found in buckets. As calabashes are plant material, the isolation and the ability for evaporation through the surface might be higher, which can buffer temperature changes of the milk. Also, the round shape of the calabashes might cause the sun to mainly warm the top of the calabash, which is not in contact with the milk, while the bottom, which had contact with the milk stays in the shadow. In case of the buckets, the sun can directly shine on the sides of the buckets, which are in contact with the milk. It seems that the colour of the buckets (white) did not help in reflecting enough sun to counter this effect.

### Acidification

During all fermentation cycles, we observed a drop in pH. The dynamics of this drop was not uniform among the fermentation cycles that received the same treatment, likely due to uncontrolled factors such as the exact timing of the start of fermentation during the day, as this depended on when the cows were milked.

Without active back-slopping, acidification had a lag phase of 6 to 18 h, both in buckets and calabashes. This variation in lag phase time could be caused by the time of the day the fermentation started. When active back-slopping was performed, we observed a strong decrease in lag time for acidification, not depending on the time of day the fermentation started. Due to the higher number of lactic acid producing bacteria present at the start of fermentation, the pH started to drop right after initiation of the fermentation process and continued during the night even when the temperature of the milk dropped. Without back-slopping the number of bacteria present in the milk is relatively low. As the temperature of the milk was going down after the start of fermentation, it took until the next day for lactic acid production to increase.

Fermentation in previously used calabashes showed great diversity in acidification rate. This is probably due to the inherent randomness of bacterial transfer from the biofilm of the calabash into the milk. For instance, the bacterial transfer can be dependent on the time between successive fermentations in the same calabash, the effectiveness of cleaning, the handling at the start of fermentation, and the physical properties of every individual calabash, such as its shape and the amount of cracks. This unpredictability of the fermentation process with passive back-slopping was also apparent in the variation in bacterial diversity over the course of fermentation. When a new calabash was used, a higher diversity was observed at the beginning of fermentation, probably due to a high influx of bacteria not associated with milk fermentation. However, the microbial community at the end of this fermentation showed a similar Shannon index compared the microbial communities of the other fermentation methods.

### Community diversity

A diverse microbial community was found in traditionally produced Mabisi. Overall, the pH decreased over fermentation time, which made the environment generally more stressful for the microbial community. This resulted in a decrease of bacterial diversity, which is probably due to selection for only those bacteria that can grow in acidic environments. As the acidification rate in the different fermentation cycles varied, also the decrease in diversity showed variation. At the end point of fermentation there was no difference in diversity anymore. However, there is an indication for a trend towards a higher diversity in Mabisi from back-slopped fermentation in buckets compared to the other methods used here.

Our method of assessment of microbial community structure relied on 16S amplicon sequencing of 444 base pairs of the gene coding for 16S ribosomal DNA (including is V4 region) that is present in (almost) all bacteria. Advantages of this method are that it allows for a comparison of bacterial communities between samples that underwent different fermentation practice, which was the main aim of the present study and for which the method generates sufficient resolution. Our method of detection does not distinguish between cells that are alive, metabolically (in)active or that are from bacteria that have died and were present at an initial or intermediate phase of fermentation. Further, while the use of DNA sequences from the V4 region allows to detect how many unique types of strains (operational taxonomic units or OTUs) are present in the community, the method does not allow for a determination to the level of species, but only mostly to the genus level. Finally, our method only detects bacteria and not eukaryotes and viruses that are also part of the microbial community. We chose to focus on bacteria when describing effects of fermentation practice in levels of backslopping on microbial community dynamics since in previous work yeast were not found to be present at high abundance in Mabisi (Moonga et al. 2020; Schoustra et al. 2013). Future work could include an analysis of all microbes (including viruses) that form the community, as well as their functionality that determine product properties.

A unique bacterial type identified as from the genus *Lactococcus* was most abundant at the end of all fermentations, between 47 and 99% of the total bacterial population. The remaining community had a very diverse character. Earlier research performed by Schoustra et al. found *L. lactis* as the main bacterial species present in the community (Chileshe et al. 2020b; Schoustra et al. 2013). However, the number of different species found in the present study is higher due to the use of a different sequencing technique. In the study by Schoustra et al (2013), a high presence of *Lactobacillus helveticus*, *Lactobacillus plantarum (*recently renamed as *Lactiplantibacillus plantarum)*, and *Acinetobacter ursingii* was found; up to 20.4%, 14.9% and 20.0%, respectively. In the current study, these species were only found in very low numbers; up to 0.4%, 0.03% and 0.09% of the total species abundance, respectively. These differences might be due to production methods of the Mabisi, sampling location, as well as methods of analysis. Unfortunately, the exact production method of the Mabisi analysed by Schoustra et al (2013) is unknown because Mabisi samples were bought from the markets. Also, a high initial growth rate of *L. lactis* might have caused it to enter the decline phase before the moment of analyses (Whiting and Cygnarowicz-Provost 1992). If this is the case, it influenced the relative frequency of *L. lactis* compared to the other bacteria.

Community diversity can be a direct consequence of the number of niches present and the availability of different organisms. According to the niche exclusion principle (Gause 1934; Hardin 1960), niches can only be occupied by one species. Consequently, an environment with more niches is more likely to have a high diversity. A niche is defined by its physical characteristics, its chemical composition, and interspecies interactions in the total bacterial community (Hutchinson 1957). Due to their smooth surface, buckets are hypothesised to have less physical niches than calabashes, which have a rough inner surface. However, on average this study did not find a lower diversity in buckets. Both vessels used can have a sufficient number of niches to maintain a diverse community, due to various characteristics.

In the experiments conducted in this study, the buckets also had a larger opening than the calabashes, and this might have caused a higher influx of environmental microbes into the fermentation media before and during fermentation. Temperatures fluctuated more in buckets compared to the calabashes most likely due to differences in the opening dimensions and heat conductivity of the materials. These fluctuations might increase the number of niches present, which could result in a more diverse community (Jiang and Morin 2007; Roxburgh et al. 2004). The milk in the containers was not mixed, increasing the possible heterogeneity in temperature and oxygen profiles, resulting again in a higher number of niches (Rainey and Travisano 1998). Besides the availability of niches in the environment, also bacterial interactions can shape niches. More research towards community function of all species, for example by the analyses of the communities on an RNA level or reconstructing communities where hypothesised key-species are deliberately left out, might give insights in the cause of diversity. Diversity may also be explained in the context of niche specialization of species over time and competition. In the calabashes, species (co-)exist for prolonged periods of time, at least longer than when growing only for one fermentation cycle as is the case in the buckets. During the prolonged growth, species may specialize on the specific niche they occupy (Lenski and Travisano 1994). Better adapted strains will outcompete the lesser adapted strains, resulting in a lower diversity in calabashes. However, when back-slopping is performed in buckets there is no such competition which can keep the diversity higher than in calabashes.

Overall an effect of the type of fermentation vessel used and the type of back-slopping on the bacterial diversity was not observed. These results do not match the trends found in recent surveys of Mabisi by Moonga et al (2019). Here, samples taken from Mabisi where some form of back-slopping was applied had a lower diversity than those without back-slopping. The fact that the back-slopping method is standard practice in that type of Mabisi might cause the difference from the current study. Repeated back-slopping will put certain selection pressures on the bacteria which changes the species composition in the communities of the Mabisi. Further studies could show the effect of back-slopping on microbial diversity as well as on aroma profile and consumer preferences, for different types of Mabisi.

### Implications of microbial diversity

Mabisi is a tasty and nutritious product and part of the cultural heritage. Therefore, understanding the microbial community can add to the production of a fermented product of high quality. The microbial community active during fermentation shapes the aroma profile of the final product as well as produces antimicrobial factors and prevents contamination.

Low pH and acidification rates have important implications for product safety. Products with a low pH are generally considered safe because this inhibits the proliferation of pathogenic bacteria (Mpofu et al. 2016; Nguz et al. 2004; Russell and Diez-Gonzalez 1997). Faster rates of acidification might also influence product characteristics, such as taste and thickness (Lucey 2004). In terms of acidification, the spontaneous fermentation of Mabisi shows similarities with fermentations performed in controlled environments using starter cultures. Moreover, the decrease in diversity is comparable to, for example, that in the production of parmesan cheese, where an undefined starter culture is mixed with raw milk and the microbial community becomes less diverse during fermentation (Gatti et al. 2014).

A microbial community with metabolic active cells of a large range of species is hypothesised to result in a product with a bigger variety of aroma components (Smid and Kleerebezem 2014) and therefore potentially a richer taste than when only few species are active. Also the chance of health beneficial bacteria to be present is higher for more diverse communities than for a product that only contains a few bacterial species (Marco et al. 2017). These bacteria can produce vitamins that are of importance to the human body or have a positive effect on the gut microbiome, which both are important for health. A more diverse community is also less prone to invasions of pathogens and bacterial spoilers (Mallon et al. 2015; Stecher et al. 2013). This, in combination with a low pH might make Mabisi a microbial safe product. With bacterial diversity alone we could not substantiate the general belief that the Mabisi produced in calabashes is different from that produced in buckets. Likely, the variation of species in the communities cause this difference.

All the effects of diverse communities mentioned above are highly relevant for the producer of a fermented product such as Mabisi because one needs a good product for home consumption as well as a product one can sell for a good price. Compared to fermented products produced using simple starter cultures with only one on two strains, Mabisi might have a clearly added benefit compared to these products, in terms of taste, nutritional value, and safety, because of its diverse bacterial community. Knowing which production steps have an influence on final product characteristics is therefore important for producers. This is true for Mabisi, but also for other products from spontaneous fermentation in general.

### Limitations

In this study we maintained the process steps as close as possible to the traditional way of producing Mabisi. While this ensures that our study aligned with actual Mabisi practice resulting in a valuable overview of what Mabisi can be and how it can be produced, it also increased the number of uncontrolled and uncontrollable variables that may influence our ability to draw firm conclusions on the effect of our treatments on the acidification and microbial community dynamics. Moreover, the number of experiments that could be performed was highly dependent on the availability of raw milk and fermentation vessels. Clearly, this had implications for the statistical power of the described experiments. The indication that active back-slopping results in a higher diversity in buckets than in calabashes might be confirmed when expanding the experimental set-up.

It is likely that besides bacteria also yeasts and fungi are members of the fermenting community in Mabisi. Our method of detection using 16S amplicon sequencing focussed on bacteria. As this is a non-alcoholic milk fermentation we chose to focus on bacteria, and particularly lactic and acetic acid bacteria, these were hypothesised to contribute most to the fermentation of Mabisi and most relevant to measure when assessing the effects of variations in fermentation practice on microbial composition. In future research, also the roles of other organisms besides bacteria should be investigated.

## Conclusions

While taking all uncontrollable variables in account, fermentation in the field showed overall great similarity to a controlled fermentation in terms of pH trajectory and microbial diversity at the end of fermentation as well as during fermentation. Bacterial communities in general decreased in diversity over time, where the drop in pH, irrespective of variation in rate of acidification, correlated with a decreasing Shannon Index. No difference was found in microbial diversity during and at the end of fermentation performed in plastic buckets or previously used calabashes. Besides small differences, all processing methods resulted in a microbial community dominated by *Lactococcus lactis* and a Shannon Index, between 0.6 and 2.0. The use of plastic buckets for Mabisi fermentation can be a valuable alternative to the use of calabashes as this study showed no biological and physico-chemical differences between Mabisi resulting from both fermentation vessels, although the reason for perceived differences should be further investigated. These results are likely to have important implications for the flavour, microbial safety, and nutritional aspects of Mabisi. Certainly, these aspects should be further investigated to meet the needs of Mabisi producers and other related products.

### Electronic supplementary material

Below is the link to the electronic supplementary material.

Electronic supplementary material 1 (XLSX 1520 kb). Bacterial composition at the level of OTUs of all samples taken and analysed. A subset of samples is shown in Figure 5 of the main text. Variables shown are time after start of fermentation (hours), vessel type and number of times a given fermentation vessel had been used for Mabisi processing.

## Figures and Tables

**Fig. 8:**
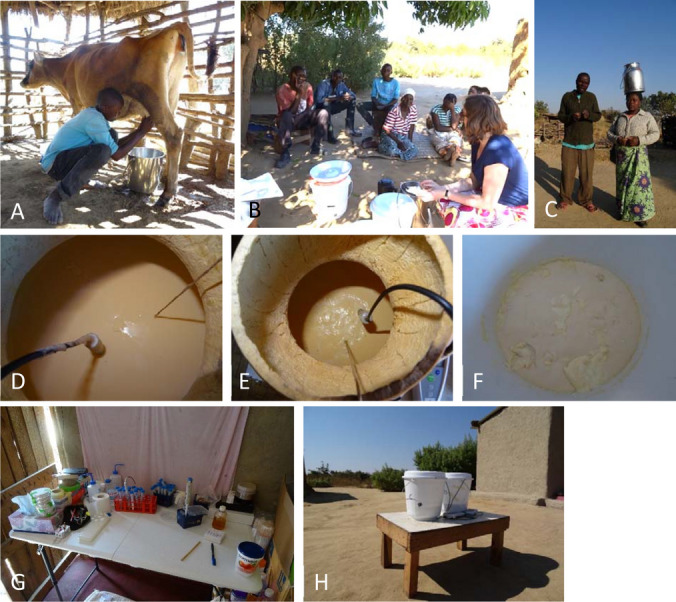
Additional pictures of the field work. Panel (A) In the early afternoon the cows are milked for the second time that day. This milk was used for the fermentations described in this study. The time of milking differed per day and per farmer and was dependent on the other activities on the farm. As the milk was originating from different farmers, also the time between milking and ‘start of fermentation’ was variable. (B) During the experiments, aspects of the study, such as pH measurements, were explained to the interested local community. (C) Milk obtained from the morning milking was brought to the milk collection centre for shipment and sale to a nationwide operating dairy company. (D & E) Temperature and pH was monitored in the fermenting milk in the calabashes. Both temperature and pH probes were positioned in the centre of the fermenting liquid. (F) Fermented Mabisi in a bucket ready for mixing and consumption. Temperature and pH probes were removed and show thickening of the milk. (G) Field laboratory for performing simple analyses and sampling. Here the samples were transferred to the filter paper, the temperature and pH probes were cleaned. (H) During the day all fermentation vessels were placed in the sun to allow temperature increase.

**Fig. 9:**
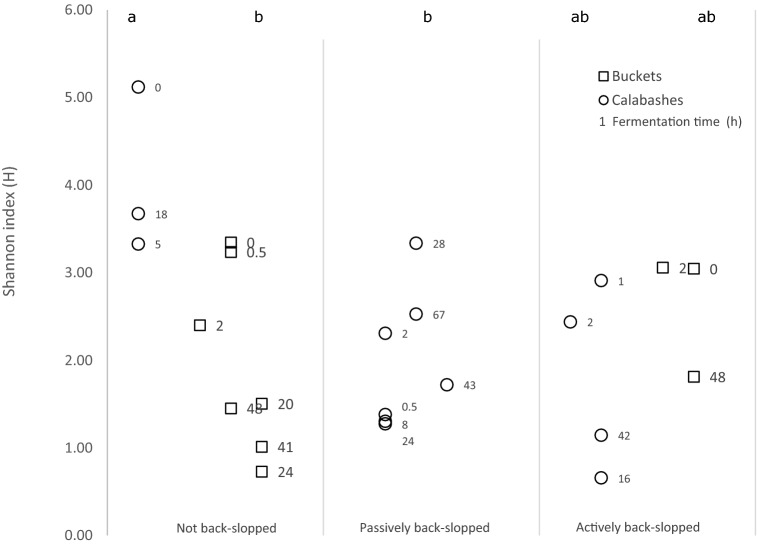
Shannon index based on OsTUs as a measure for bacterial diversity during fermentations in buckets (open square) and calabashes (open circle) with and without back-slopping. Number next to data points indicate time after start of fermentation in hours. Different fermentations from one processing method presented next to each other. Letters above the columns indicate significant difference at the 0.05 level taking all samples from one method together. Some samples could not be included because the field experiment did not allow regular sampling and some samples were lost due to contamination

## Appendix A

See Fig. 8.

## Appendix B: Information about Zambia and the importance of Mabisi

In Zambia over 400 dairy cooperatives exist. The Mondake Dairy Association based in the rural part of Kabwe town in Zambia is a typical local farmer organisation. The Association expressed that local milk-processing and sales would present excellent opportunities for women entrepreneurship and financial self-reliance for women and their households. The Association has adopted an active policy towards improved gender equality and women’s leadership.

Five years ago, the Association had around 100 members, of whom 20% were women. Currently, the membership has increased to 1274 and 60% are women. Members own 7 cows on average and milk 3 of their 7 cows, producing about 15 lper day. Combined, the members of the Association produce 900 l of milk per day. Around 75% of raw milk on a daily basis, especially the afternoon milk, is not collected at the milk collection centre by the commercial dairy company. The uncollected raw milk spoils and is discarded, leading to high postharvest losses at the very source of the milk value chain. Furthermore, the 900 lare produced from milking only half of the available cows, as milking more cows would only increase the loss of fresh milk. Around 10% of milk is used to make *Mabisi* for household consumption, and excess *Mabisi* is sold to local customers. Fresh milk of grade A sells at the local rural market or commercial milk collection centre, at 4 kwacha per cup (EUR 0.25) and *Mabisi* at 10 to 15 kwacha per cup (EUR 1 to 1.5).

Currently, there is no option to larger scale *Mabisi* production due to lack of facilities, training, and the lack of scaling up of technology. Members of the Association have been part of a project on traditional Mabisi. This project has generated basic knowledge on processing standards, on which the Association organised a workshop for its members jointly with the University of Zambia, Heifer International and Wageningen University. Furthermore, one of the members has visited the Netherlands for a two-week workshop on dairy processing in the Netherlands, hosted at Wageningen University. This trip has been highly motivating and stimulating for the entire Association. The Cooperative is now looking into how to adopt new technology as well as develop skills in business development and marketing to transform themselves in a cooperative that can be an equal partner to other players in the value chain of agricultural produce.

## Appendix C

See Fig. 9. Bacterial composition at the level of OTUs of all samples taken and analysed. A subset of samples is shown in Figure 5 of the main text. Variables shown are time after start of fermentation (hours), vessel type and number of times a given fermentation vessel had been used for Mabisi processing.
